# The role of ultrasound in enhancing mesenchymal stromal cell‐based therapies

**DOI:** 10.1002/sctm.19-0391

**Published:** 2020-03-10

**Authors:** Daniel D. Liu, Mujib Ullah, Waldo Concepcion, Jeremy J. Dahl, Avnesh S. Thakor

**Affiliations:** ^1^ Interventional Regenerative Medicine and Imaging Laboratory, Department of Radiology Stanford University Palo Alto California; ^2^ Department of Surgery Stanford University Palo Alto California

**Keywords:** cell therapy, extracorporeal shockwave therapy, focused ultrasound, homing, low‐intensity ultrasound, mesenchymal stromal cells, regenerative medicine, ultrasound

## Abstract

Mesenchymal stromal cells (MSCs) have been a popular platform for cell‐based therapy in regenerative medicine due to their propensity to home to damaged tissue and act as a repository of regenerative molecules that can promote tissue repair and exert immunomodulatory effects. Accordingly, a great deal of research has gone into optimizing MSC homing and increasing their secretion of therapeutic molecules. A variety of methods have been used to these ends, but one emerging technique gaining significant interest is the use of ultrasound. Sound waves exert mechanical pressure on cells, activating mechano‐transduction pathways and altering gene expression. Ultrasound has been applied both to cultured MSCs to modulate self‐renewal and differentiation, and to tissues‐of‐interest to make them a more attractive target for MSC homing. Here, we review the various applications of ultrasound to MSC‐based therapies, including low‐intensity pulsed ultrasound, pulsed focused ultrasound, and extracorporeal shockwave therapy, as well as the use of adjunctive therapies such as microbubbles. At a molecular level, it seems that ultrasound transiently generates a local gradient of cytokines, growth factors, and adhesion molecules that facilitate MSC homing. However, the molecular mechanisms underlying these methods are far from fully elucidated and may differ depending on the ultrasound parameters. We thus put forth minimal criteria for ultrasound parameter reporting, in order to ensure reproducibility of studies in the field. A deeper understanding of these mechanisms will enhance our ability to optimize this promising therapy to assist MSC‐based approaches in regenerative medicine.

## Graphical abstract

Mesenchymal stromal cells (MSCs) are a promising platform for regenerative medicine, but their therapeutic efficacy still requires optimization. Ultrasound (US) can enhance MSC‐based therapies through a variety of strategies. When applied to MSCs in vitro, US modulates self‐renewal and differentiation. When applied to target organs in vivo, US enhances MSC homing by upregulating local cytokines and adhesion molecules.

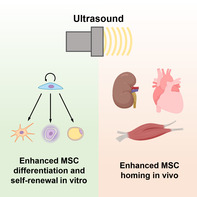

**Significance statement**

Mesenchymal stromal cells (MSCs) are a popular platform for regenerative medicine due to their ability to home to damaged organs and secrete molecules that spur cell growth and suppress inflammation. However, there remains a need to optimize their therapeutic effect for clinical translation. One such strategy is the use of ultrasound. Ultrasound can be applied to MSCs to enhance their ability to secrete regenerative molecules or applied to a target organ to make it a more attractive destination for infused MSCs. The present article reviews the current knowledge of ultrasound's biological effects and preclinical applications for MSC‐based therapies.

## 1 MESENCHYMAL STROMAL CELL BIOLOGY

Within the field of regenerative medicine, mesenchymal stromal cells (MSCs) have been a popular area of research owing to their anti‐inflammatory effects, secretion of growth factors, and ability to home to damaged tissue.^1^, ^2^ MSCs are multipotent cells that, as their name suggests,^2^ can give rise to various mesenchymal lineages, including bone, cartilage, and adipose tissue. Though they were first isolated from bone marrow,^3^ MSCs have since been purified from a variety of other tissues, including adipose,^4^ muscle, dermis,^5^ dental pulp,^6^ perivasculature,^7^ and Wharton jelly from the umbilical cord.^8^, ^9^

MSCs are believed to play a natural regenerative role in the human body: in response to tissue damage, MSCs are released into circulation, where they home to the site of injury in response to inflammatory signals.^2^ MSC homing is a multistep process which can be split into five steps: (a) tethering and rolling, (b) activation, (c) arrest, (d) transmigration/diapedesis, and (e) nonsystemic migration.^10^ During *tethering*, CD44 expressed on the MSC surface catch onto selectins on the endothelium, after which they begin *rolling* along the vessel wall.^11^ *Activation* is facilitated by G‐protein coupled chemokine receptors, most prominently CXCR4, which binds stromal cell‐derived factor 1 (SDF‐1) released by inflamed tissue.^12^ These interactions activate integrins (VLA‐4) on the MSC surface, which then bind to receptors on the endothelium (VCAM‐1) to trigger cell *arrest*. After arrest, MSCs undergo *transmigration or diapedesis* to pass through the endothelium. This step is facilitated by the secretion of enzymes like matrix metalloproteinases (MMPs) that break down the endothelial basement membrane.^13^ Finally, having exited the systemic circulation, MSCs undergo further *nonsystemic migration* to reach the injured tissue, guided by chemokines and growth factors.^14^ Within the tissue, they secrete a variety of factors with powerful immune‐modulating, angiogenic, and antiapoptotic effects.^15^, ^16^, ^17^ MSCs are highly immunosuppressive, being able to convert pro‐inflammatory environments into anti‐inflammatory environments by suppressing T cell, B cell, natural killer (NK) cell, and dendritic cell populations, as well as by expanding regulatory T‐cell pools.^18^ Their angiogenic ability is also well documented, owing to their ability to secrete potent angiogenic factors like vascular endothelial growth factor (VEGF), insulin‐like growth factor 1 alpha, and hepatocyte growth factor (HGF),^19^ which activate the PI3K‐Akt pathway in endothelial cells to inhibit apoptosis, increase survival, and stimulate new blood vessel formation.^20^

Given these regenerative abilities, there has been great interest in exploiting the therapeutic potential of MSCs. MSCs can be cultured in vitro and then transfused into patients, after which they home to damaged tissue to aid in recovery and serve as an effector for tissue regeneration.^1^ Several properties make them attractive platforms for cell‐based therapy. They are easy to harvest from bone marrow or adipose tissue, expand in culture, and can then be transplanted into patients via an intravenous injection. MSCs appear to be somewhat immune‐privileged,^21^, ^22^, ^23^ and many clinical trials have demonstrated their safety in humans. Indeed, there are over 100 registered clinical trials using MSCs for applications such as immune modulation in multiple sclerosis and type 1 diabetes, tissue protection following myocardial infarction or liver cirrhosis, and tissue regeneration for bone and cartilage repair.^24^ The results of such trials, though promising, leave much room for improvement. Perhaps the biggest hurdle encountered by MSCs is their ability to be targeted to their intended destination. When MSCs are infused intravenously, only a few percent ultimately reach the target tissue due to inefficient homing.^25^ Another hurdle is stimulating the MSCs to secrete regenerative factors in sufficient quantities once they reach the damaged tissue, in order that they have an appreciable clinical effect.

Many strategies have been used to improve the homing and regenerative capabilities of MSCs, including genetic modification, cell surface engineering, and in vitro priming.^26^, ^27^, ^28^ One novel method for improving MSC‐based therapies comes in the form of ultrasound, which has been shown to be effective both for improving MSC homing and their regenerative capabilities. This review discusses ultrasound‐based methods that have been demonstrated to enhance MSC‐based therapies and the potential molecular mechanisms by which they do so.

## 2 THERAPEUTIC ULTRASOUND

Although ultrasound is most commonly used for diagnostic imaging, it has been adopted for a variety of therapeutic applications since the 1950s.^29^ Therapeutic ultrasound often utilizes acoustic pressures and intensities well above those of diagnostic ultrasound (DUS) in order to elicit some form of biological effect or response. Typically, the ultrasound beam is focused to a point within the body, thereby selectively targeting a specific tissue of interest and avoiding bioeffects in the tissues lying between the ultrasound transducer and the target tissue.

### 2.1 Forms of therapeutic ultrasound

**Figure 1:**
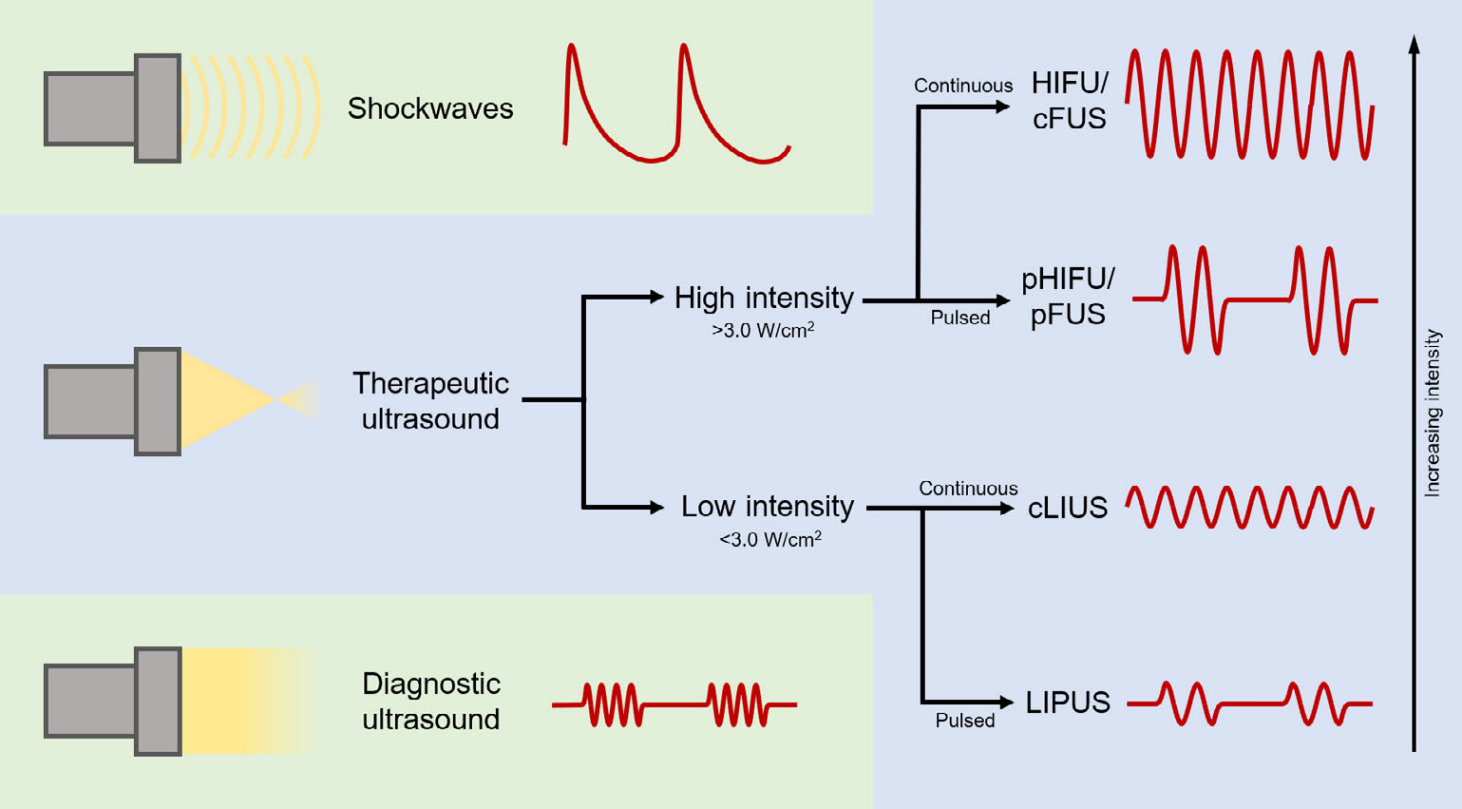
Ultrasound modalities. Schematic of different forms of ultrasound that have been used for enhancing MSC‐based therapies, along with representative waveforms. Intensity values reflect ranges typical of studies in the literature. cFUS, continuous focused ultrasound; cLIUS, continuous low‐intensity ultrasound; HIFU, high‐intensity focused ultrasound; LIPUS, low‐intensity pulsed ultrasound; MSC, mesenchymal stromal cell; pFUS, pulsed focused ultrasound; pHIFU, pulsed high‐intensity focused ultrasound

Under the umbrella of ultrasound therapy, a variety of methods have been investigated, with different modes of delivery, intensity, and biological mechanisms. A few specific examples of therapeutic ultrasound include high‐intensity focused ultrasound (HIFU) for tissue and tumor ablation,^30^ histotripsy (the mechanical fractionation of tissue) to break up and liquefy diseased tissue,^31^ low‐intensity pulsed ultrasound (LIPUS) for aiding bone fracture healing,^32^ and extracorporeal shockwave therapy (ESWT) for breaking kidney and bladder stones.^33^ Even DUS has also been used in therapeutic contexts, though always in conjunction with adjuvants. Adjuvants are agents used to amplify the effect of ultrasound: in ultrasound‐mediated microbubble destruction (UMMD), microscopic bubbles are injected into the bloodstream, and upon exposure to focused ultrasound, the bubbles cavitate to cause a variety of physical and biological effects. Researchers have broadly categorized ultrasound, into low vs high‐intensity and continuous vs pulsed methodologies (Figure 1). The labels, however, are arbitrary and often inconsistent. For organizational purposes, we will keep the labels as reported in the literature. However, these different forms of ultrasound can be better described using a spectrum of intensities and other parameters.

### 2.2 Ultrasound parameters

**Figure 2:**
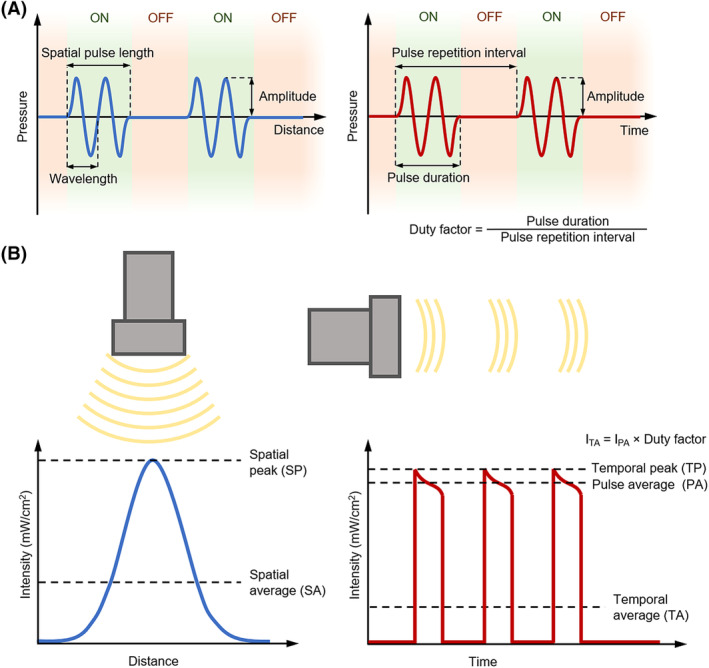
Ultrasound parameters. A, Representations of various parameters related to pulsed ultrasound, with waveforms represented over space (left) or time (right). B, Representations of the various measures of intensity in pulsed ultrasound. Left, the spatial intensity of a beam of ultrasound over its cross‐sectional area, showing the spatial average and peak. Right, the temporal intensity of several pulses of ultrasound, showing the temporal average and peak, and pulse average

To understand the various forms of therapeutic ultrasound discussed in this review, we will briefly review their basic physical parameters. Ultrasound frequency is the number of times per second a particle experiences a complete compression and rarefaction cycle, and is given in units of hertz (Hz) or 1/second. Low‐frequency (20‐200 kHz) and medium‐frequency (0.7‐3.0 MHz) ultrasound have generally been used for therapeutic purposes, whereas high‐frequency ultrasound (1‐20 MHz) is generally used for imaging and diagnostics.^34^ In pulsed ultrasound, the transducer administers small pulses of waves that are temporally separated. The ratio of time that the transducer is “on” to the total time between the start of the pulses (ie, time “on” plus time “off”) is called the duty cycle. A duty cycle of 100% means that the transducer is continuously transmitting, a form of ultrasound called “continuous wave” ultrasound. The number of pulses transmitted per second is referred to as the pulse repetition frequency (PRF), given in hertz. These parameters are illustrated in Figure 2A. The potential for generating bioeffects and determining safety is often based on the intensity of the transmitted ultrasound. Intensity is the rate at which energy is deposited per unit area, often given in units of watts (W) or milliwatts (mW) per square centimeter (eg, mW/cm^2^). Because intensity varies both temporally (with the “on” and “off” nature of pulses) and spatially (since the edge of an ultrasound beam is less intense than its center), there are various ways to describe intensity. The two common measures of spatial intensity are spatial average (SA) and spatial peak (SP), which are the average and maximum intensities over the cross‐sectional area of an ultrasound beam, respectively (Figure 2B). There are three common measures of temporal intensity: temporal average (TA) and temporal peak (TP), which take the average and maximum intensity over time, respectively, and pulse average (PA), which takes the average just when the transducer is on. This makes for a total of six combinations of temporospatial intensities (SATA, SAPA, SATP, SPTA, SPPA, SPTP). The mechanical index (MI) indicates the likelihood of causing a mechanical bioeffect, such as cavitation, and is defined as the peak negative pressure of the ultrasound wave divided by the square root of its frequency.

In this review, we focus specifically on those forms of therapeutic ultrasound that have been tested in conjunction with MSC‐based therapies. These strategies can be broadly categorized into two approaches. First are the ones that apply ultrasound to the target tissue, upregulating the expression of homing factors so as to make it a more attractive target for MSCs (Table 1). Second are the ones that apply ultrasound to cultured MSCs in vitro, so as to modulate their self‐renewal, differentiation, and production of regenerative factors (Table 2). These approaches have been applied to a variety of organ systems and disease models (Table 3).

**Table 1:** Genes modulated by ultrasound in sonicated tissue in vivo

	Gene	DUS	LIPUS		pFUS		ESWT
		+MB	−MB	+MB	−MB	+MB	
Cytokines	*BMP2*						[35]
	*CCL2/MCP‐1*	[36]			[37, 38, 39, 40, 41, 42, 43]	[44, 45]	
	*CCL3/MIP‐1α*				[37, 39, 40, 41, 43]		
	*CCL4/MIP‐1β*				[40]		
	*CCL5/RANTES*				[37, 38, 39, 40, 41, 43]	[45]	[35]
	*CCL11/Eotaxin‐1*				[40]	[45]	
	*CCL12*					[45]	
	*CCL20/MIP‐3α*					[45]	
	*CCL22*					[45]	
	*CSF1/M‐CSF*				[39, 40, 42, 43]	[45]	
	*CSF2/GM‐CSF*				[37, 38, 40, 41]	[45]	
	*CSF2RB*					[45]	
	*CSF3/G‐CSF*				[40, 43]	[45]	
	*CXCL1/GRO1*				[39, 40, 43]	[45]	
	*CXCL2/MIP‐2*				[39, 40]		
	*CXCL3*					[45]	
	*CXCL9/MIG*				[39, 40, 43]	[45]	
	*CXCL10/IP‐10*				[40, 42]	[45]	
	*CXCL12/SDF‐1α*	[36]	[46]		[37, 39, 41]	[47, 48, 49, 50]	[51, 52, 53, 54, 55, 56]
	*IFN‐γ*	[36]			[37, 38, 40, 41, 42]		
	*IL‐1α*	[36]			[40, 41, 43, 57]	[45]	
	*IL‐1β*			[58]	[37, 38, 39, 40, 41, 42, 43]	[44, 45, 47, 50]	[35, 59]
	*IL‐1R2*					[45]	
	*IL‐1RA*					[45]	
	*IL‐2*	[36]			[38, 39, 40, 42]		
	*IL‐3*	[36]			[37, 38, 39, 40]		
	*IL‐4*				[37, 40, 43]	[44, 45]	
	*IL‐5*				[37, 38, 40, 43]		
	*IL‐6*				[38, 39, 40, 42]	[44, 45]	
	*IL‐9*				[37, 39]		
	*IL‐10*				[37, 38, 39, 40, 43]		
	*IL‐12p40*				[37, 39, 43]		
	*IL‐12p70*				[40, 43]		
	*IL‐13*				[39, 40, 43]	[45]	
	*IL‐15*				[39, 40, 43]		
	*IL‐17*				[38, 39, 43]	[45]	
	*IL‐18*				[40]	[45]	
	*IRF1*					[45]	
	*LIF*				[39, 40]		
	*SCF*				[37]		
	*TNF‐α*	[36]		[58]	[37, 39, 40, 42, 57]	[44, 45]	[35, 53, 59]
	*TNFR2*					[45]	
	*TGF‐β*				[37, 42]		[35, 60]
Growth factors	*EGF*				[40]	[61]	
	*EPO*				[40]	[45]	
	*FGF*				[38, 40, 41, 43]	[45, 62]	
	*HGF*				[37, 38, 41, 43]	[61]	
	*IGF‐1*				[39]		
	*PDGF*				[39, 40]		
	*PLGF*				[38, 41]		
	*VEGF*	[36, 63]			[37, 38, 39, 40, 41, 42, 43]	[45, 47, 48, 50, 62]	[51, 52, 53, 56, 60]
Adhesion molecules	*ICAM‐1*				[37, 38, 40, 41, 43]	[61, 62]	[35]
	*LAM*					[44]	
	*PECAM*						[52, 53, 56]
	*SELE*	[36]				[45]	
	*SELEP*					[45]	
	*VCAM‐1*	[36, 63]			[37, 38, 39, 40, 41, 43]	[49, 50, 62]	
	*vWF*						[52]
Matrix remodelers	*MMP9*					[45]	[35, 52, 53, 59]
	*PLAU*					[45]	
Immune	*C3*					[45]	
	*CD40*					[45]	
	*CD83*					[45]	
	*COX2*				[39, 40, 57, 64]		
	*NFKB*				[39, 40, 57]	[45]	[35, 53, 59]
	*NFKBIA*					[45]	
	*STAT3*					[45]	
	*TLR2*						[35]
	*TLR4*						[35]
Endocrine	*ADM*					[45]	
	*AGT*					[45]	
	*INS2*					[45]	
	*PTGS2*					[45]	
Ion channels	*TRPC1*				[64]		
	*VGCC*						
Apoptosis	*BAX*						[52, 53, 59]
	*BCL2A1*					[45]	[35, 52]
	*BIRC3*					[45]	
	*Caspase‐3*						[35, 52, 53, 59]
	*PARP*						[35, 53, 59]
Oxidative stress	*Cytochrome C*						[35, 52, 59]
	*NOX1*						[35, 53, 59]
	*NOX2*						[35, 53, 59]
	*NT‐proBNP*				[40]		
	*PGC‐1α*						[52]
	*pH2AX*						[35, 59]
	*SIRT1*						[59]
	*SIRT3*						[59]
	*eNOS*						[52, 53]
Other transcription factors	*EGR2*					[45]	
	*MYC*					[45]	
	*NR4A2*					[45]	

Abbreviations: DUS, diagnostic ultrasound; ESWT, extracorporeal shockwave therapy; LIPUS, low‐intensity pulsed ultrasound; MB, microbubble; pFUS, pulsed focused ultrasound.

**Table 2:** Genes modulated by ultrasound in mesenchymal stromal cells (MSCs) in vitro

		Gene	LIUS	HIFU		ESWT
				−MB	+MB	
Adhesion		*CD29/Integrin β1*	[65, 66, 67]			
		*CD44*	[66]			
		*CX43*	[68]			[69]
		*ICAM‐1*			[49]	
		*VCAM‐1*			[49]	
Cytokines		*CCR2*	[65]			
		*CXCL5*				[70]
		*CXCL12/SDF‐1α*	[46]		[49]	[53, 71]
		*CXCR4*	[46, 65]	[48]	[48, 49]	[53]
		*IL‐8*	[72]			
Proliferation		*Cyclin A2*	[73]			
		*Cyclin B1*	[73]			
		*Cyclin D1*	[73, 74]	[75]		
		*Cyclin E1*	[73]			
Growth factors		*ANGPT*				[53]
		*BDNF*	[76]			
		*NGF*	[76]			[71]
		*VEGF*	[72]			[53, 56, 60, 70, 71, 77]
ECM		*MMP13*	[78]			
		*TIMP2*	[78, 79]			
Differentiation	Stem	*Nanog*	[80]			
	Liver	*AFP*		[75]		
		*ALB*		[75]		
		*CK18*		[75]		
	Bone	*ALP*	[66, 72, 81, 82, 83, 84, 85]			[86, 87]
		*BMP2*	[82, 85]			[77]
		*CBFA1*	[83]			
		*COL1*	[66, 67, 72, 78, 82, 85]			[87]
		*COL10*	[78]			
		*miR‐138*				[88]
		*OCN/BGLAP*	[66, 72, 82, 83, 85, 89]			
		*OPG*	[83]			
		*OPN/SPP1*	[72, 82, 85, 90]			
		*OSX*	[83, 90]			[87]
		*RUNX2*	[81, 82, 85, 89]			[87, 88]
	Cartilage	*Aggrecan*	[67, 78, 79, 81, 91]			
		*COL2*	[67, 79, 81, 92, 93]			
		*SOX9*	[67, 78, 79, 81]			
	Adipose	*APN*	[94]			
		*FABP4*	[89]			
		*PPARγ*	[89, 94]			
	CNS	*CACNA1*	[95]			
		*MAP2*	[95]			
		*ND1*	[95]			
		*Nestin*	[95]			
		*NF‐L*	[95]			
		*Tau*	[95]			
Signaling		*β‐Catenin*		[75]		
		*cMyc*		[75]		
		*FAK*				[88]
		*MAP3K8/COT/TPL2*	[89]			
		*MAPK/ERK*	[72, 73, 74, 82, 89]			[69]
		*mTOR*	[67]			
		*PI3K/AKT*	[73, 74]			
		*RANKL*	[83]			
		*ROCK*	[89]			
		*P2Y receptor*	[68]			
		*TGFβ*				[77, 86]
		*WNT1*		[75]		

Abbreviations: ESWT, extracorporeal shockwave therapy; HIFU, high‐intensity focused ultrasound; LIUS, low‐intensity ultrasound; MB, microbubble.

**Table 3:** Studies applying ultrasound to mesenchymal stromal cells (MSCs) or MSC‐based therapies, organized by organ system

Organ system	DUS	LIUS		HIFU		ESWT
	+MB	−MB	+MB	−MB	+MB	
Adipose		[89, 94]				
Bone		[46, 66, 72, 81, 82, 83, 84, 85, 89, 90, 96, 97, 98]				[60, 77, 86, 87, 88, 99]
Cartilage		[67, 78, 79, 81, 91, 92, 93, 98, 100]				
CNS		[76, 95]			[45, 101]	[54, 59]
Heart	[63, 102]			[40]	[44, 47, 48, 49, 50, 62, 103]	[52, 53]
Kidney	[36]			[38, 57, 64, 104]	[61, 105]	
Liver				[75]		
Muscle				[37, 39, 41, 43, 64]		[35, 51]
Pancreas				[42]		
Urogenital			[58]			[55, 56, 71]
Undifferentiated		[65, 68, 73, 74, 80]				[69, 70]

Abbreviations: CNS, central nervous system; DUS, diagnostic ultrasound; ESWT, extracorporeal shockwave therapy; HIFU, high‐intensity focused ultrasound; LIUS, low‐intensity ultrasound; MB, microbubble.

## 3 PULSED FOCUSED ULTRASOUND

Pulsed focused ultrasound (pFUS), sometimes referred to as pulsed high intensity focused ultrasound, is a therapeutic ultrasound method that uses short‐duration, high‐intensity pulses to nondestructively target tissues of interest. Though there is wide variation in the parameters that constitute pFUS, many of the studies discussed in this section report I_SATA_ = 133 W/cm^2^, PRF 5 Hz at 5% duty cycle, frequency 1 MHz. pFUS has been shown to be relatively safe, causing minimal histological alterations.^37^, ^38^ Although one study found enlarged gaps between muscle fiber bundles following pFUS sonication, these differences went away within 72 hours.^106^ Although HIFU, also known as continuous focused ultrasound (cFUS), generates extreme temperatures to ablate tissue, pFUS avoids tissue damage and temperature elevation.^37^ Indeed, heat shock protein‐70, which is strongly upregulated by heat stress, does not appear to increase following pFUS.^37^, ^39^, ^40^, ^104^ Instead, pFUS primarily elicits mechanical stimulation of the tissue, which upregulates inflammatory and other chemoattractive molecules. These molecular changes are short‐lived, lasting only around 24‐36 hours,^38^ enough time to promote MSC homing to the sonicated area. Importantly, the increased homing following pFUS seems to result not from increased leakiness of the vasculature but rather from the induced molecular changes.^38^

### 3.1 Molecular mechanism of pFUS‐mediated MSC homing

**Figure 3:**
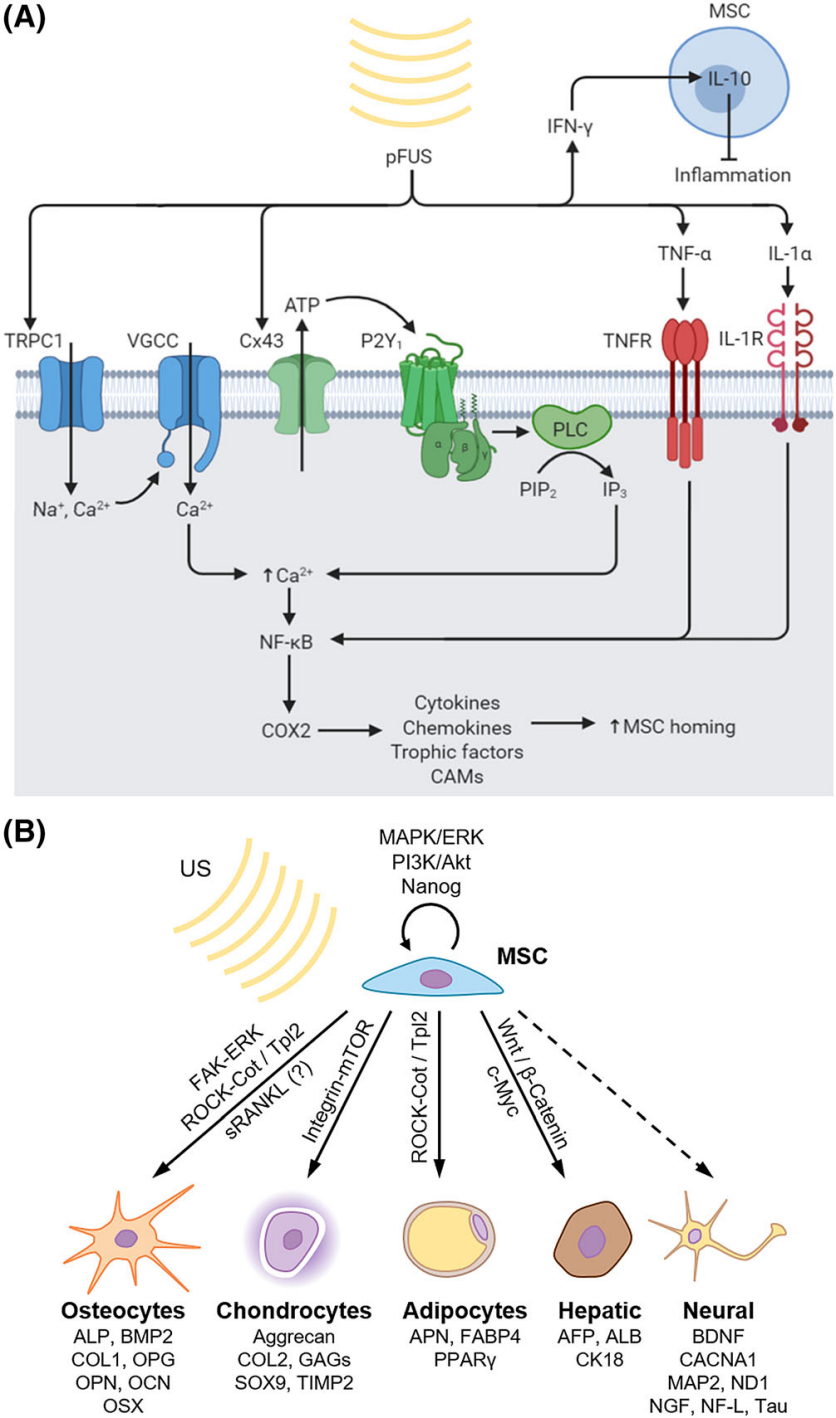
Molecular mechanisms of ultrasound. A, Currently known mechanisms by which pulsed focused ultrasound (pFUS) enhances mesenchymal stromal cell (MSC) homing in vivo. B, The effect of low‐intensity pulsed ultrasound (LIPUS) and extracorporeal shockwave therapy (ESWT) on MSC proliferation and differentiation in vitro. Upregulated markers are listed underneath each lineage. Involved signaling pathways are shown along the arrows

Research on the molecular mechanisms underlying pFUS and its therapeutic potential, though scant, has been gaining steady interest (Figure 3A). Burks et al conducted one of the first systematic investigations into the molecular response to pFUS,^41^ using the murine hamstring muscle as the target organ. Their results demonstrated that pFUS, unlike cFUS, did not affect histological integrity of the muscle and did not induce apoptosis. It did, however, create a local cytokine gradient lasting for 3 days, along with the upregulation of signaling molecules (SDF‐1α, IL‐1α, IL‐1β, MCP‐1, IFNγ, MIP‐1α, GM‐CSF, RANTES), growth factors (VEGF, FGF, HGF, PLGF), and cell adhesion molecules (ICAM‐1, VCAM‐1) on the endothelium. A follow‐up study verified these molecular changes,^37^ and further demonstrated that the pFUS‐induced cytokine gradient enhanced homing, permeability, and retention of MSCs to the murine hamstring, around 4.5‐fold higher in treated muscle compared with control groups at 24 hours post‐injection. Homing was further improved with repeated administration of pFUS and MSC infusions. After three daily doses of combined pFUS and MSCs, homing was increased nearly fivefold compared with the single dose treatment. pFUS was also shown to induce an anti‐inflammatory M2 macrophage response, whereas cFUS induced a pro‐inflammatory M1 response. Work by Tebebi et al uncovered some of the molecular pathways underlying the mechanotransduction elicited by pFUS.^39^ They showed via proteomic analysis that the tumor necrosis factor alpha (TNF‐α) is one of the first genes activated following pFUS to the murine hamstring (around 10 minutes post‐treatment). TNF‐α was shown to drive NK‐κB and subsequently cyclooxygenase‐2 (COX2) activity, which in turn was responsible for the upregulation of homing factors. Additionally, pFUS was found to mechanically open the TRPC1 cation channel on the plasma membrane, causing an influx of sodium and calcium that depolarizes the membrane and activates the voltage‐gated calcium channel (VGCC), causing further calcium influx which activates NF‐κB and subsequently COX2.^64^ Indeed, pFUS failed to increase MSC homing to the muscle when administered alongside ibuprofen (a COX inhibitor) or etanercept (a TNF‐α inhibitor), as well as in COX2‐knockout mice.^39^

Similar results have been achieved in the kidney. Ziadloo et al exposed murine kidneys to pFUS and demonstrated a similar change in gene expression as in muscle, including increases in cytokines (IL‐1β, IL‐2, IL‐3, IL‐5, IL‐6, IL‐10, IL‐17, IFNγ, MCP‐1, GM‐CSF, RANTES), growth factors (VEGF, FGF, HGF, PLGF), and cell adhesion molecules (ICAM‐1, VCAM‐1), with expression levels returning to baseline after 3 days.^38^ pFUS alone had no effect on renal function (measured by blood urea nitrogen and serum creatinine) or changes in renal architecture, and no evidence of increased apoptosis, hemorrhage, or necrosis. However, pFUS increased the homing of MSCs to the kidney, eightfold on day 1 and fivefold on day 3, with this difference disappearing after day 7. While pFUS increased the expression of cytokines in the kidney, the combination of pFUS and MSCs did not, reflecting the latter's anti‐inflammatory effect. Importantly, there was no evidence of extravascular red blood cells (RBCs); since RBCs are smaller than MSCs, their absence outside the vasculature demonstrates that the increased MSC homing was not due to increased leakiness of the vessels but rather due to molecular changes induced by pFUS. Akin to muscle, pFUS caused early elevations in TNF‐α and IL‐1α in the kidney, driving molecular responses through NF‐κB‐ and COX2‐dependent pathways that activate cytokines, trophic factors, and cell adhesion molecules.^57^ Similar to muscle, pFUS to the kidney mechanically opens TRPC1 cation channels, which in turn opens voltage‐gated VGCC receptors; the ensuing calcium influx activates NF‐κB which drives COX activity.^64^ Indeed, pFUS failed to increase kidney homing when paired with ibuprofen (a COX inhibitor), etanercept (a TNF‐α inhibitor), anakinra (an IL‐1 receptor antagonist), or prednisone (an NF‐κB translocation inhibitor), or when administered to COX2‐knockout mice.^57^

One study investigated the effect of pFUS on the native pancreas.^42^ Here, pFUS to the pancreas had no effect on tissue histology and did not elevate serum amylase or lipase (markers of pancreatitis). Interestingly, the study found a differential effect between lower (11.5 W/cm^2^) vs higher (18.5 W/cm^2^) pFUS intensities. Lower intensity pFUS downregulated growth factors (M‐CSF, TGFβ, VEGF) and pro‐inflammatory cytokines (IL‐1β, IL‐2, IL‐6, IFN‐γ, IP‐10, TNF‐α), whereas higher intensity pFUS upregulated growth factors (MCP‐1, TGF‐β) and pro‐inflammatory cytokines (IL‐1β, IFN‐γ, TNF‐α). Though this study did not measure MSC homing, this differential effect highlights that pFUS parameters can greatly influence its bioeffects and need to be finely tuned based on the desired application.

Based on these studies, a rudimentary understanding of pFUS‐induced MSC homing is beginning to emerge (Figure 3A). However, much remains unknown regarding the complete molecular mechanism and the involved signaling pathways. Different intensities and parameters of pFUS may also elicit different tissue responses, a question that is only beginning to be rigorously explored.

### 3.2 Application of pFUS to disease models

Tebebi et al tested pFUS + MSC combination therapy on a mouse model of critical limb ischemia.^43^ They found that the combined treatment resulted in a fourfold increase in MSC homing compared with MSCs alone, even 5 weeks post‐injection. It also increased vascular density (measured by CD31 count) by twofold and decreased the fibrotic area by ~50%. In addition, pFUS treatment altered gene expression on the MSCs themselves: MSCs administered in combination with pFUS expressed more VEGF and IL‐10 (threefold and fourfold, respectively) compared with those administered without pFUS.

Burks et al tested pFUS + MSC therapy on a mouse model of cisplatin‐induced acute kidney injury (AKI) to demonstrate its clinical potential.^104^ They showed that pFUS + MSCs enhanced homing to the injured kidney (~1.4‐fold), improved outcomes of renal function (65% reduced blood urea nitrogen and 80% reduced serum creatinine), prevented apoptosis (60%) and necrosis (80%) in the tubules, and promoted regeneration significantly more compared with MSCs only. The combined treatment improved survival and kidney function even in late therapy (3 days after cisplatin treatment), after renal function had already declined. A follow‐up study elucidated the underlying mechanism in the AKI model: pFUS upregulates IFNγ in the injured kidney, which stimulates MSCs to produce IL‐10, an anti‐inflammatory cytokine that promotes recovery.^107^ They also demonstrated that IFNγ stimulation upregulates IL‐10 in MSCs in vitro and improves AKI outcomes more so than unstimulated MSCs. Indeed, pFUS failed to improve AKI outcomes either if the mice were IFNγ‐deficient or if the MSCs were IL‐10‐deficient.

Several studies have been conducted on the effects of pFUS on the heart, though most have utilized microbubbles,^44^, ^47^, ^62^ which are discussed below. When targeted to infarcted regions of the canine myocardium, pFUS with microbubble injection increased expression of IL‐1β, VEGF, VCAM‐1, and SDF‐1α.^47^ In rat hearts, a similar treatment elevated the protein levels of IL‐1β, IL‐4, IL‐6, MCP‐1, and TNF‐α, although not VEGF.^44^ At least one study has investigated the molecular changes following pFUS without microbubble destruction.^40^ Using a rat model, Jang et al observed a gene expression pattern in the heart following pFUS similar to those in the muscle and kidney: an initial increase in TNF‐α followed by the upregulation of both pro‐ and anti‐inflammatory cytokines (IL‐1α, IL‐1β, IL‐2, IL‐4, IL‐5, IL‐13, G‐CSF, GM‐CSF, MIP‐1α, IFN‐γ, MCP‐1, IP‐10, GRO‐KC, RANTES) and growth factors (EGF), though again, VEGF was not increased. These molecular changes returned to baseline after 24 hours. However, they also observed a brief but significant increase in the cardiac injury marker NT‐proBNP, and the use of higher intensity pFUS (>4 MPa) caused myocardial edema and pulmonary hemorrhage. Effects on MSC homing efficiency were not measured.

## 4 LOW‐INTENSITY ULTRASOUND

Low‐intensity ultrasound (LIUS), as its name suggests, is administered at low intensities. The reported values that constitute “low intensity” are generally in the range of I_SATA_ = 0.03‐3 W/cm^2^.^108^ Although there is no consistency in the values reported, the “prototypical” parameters are I_SATA_ 30 mW/cm^2^, PRF 1000 Hz at 20% duty cycle, and frequency 1.5 MHz.^34^ However, the definition of “low intensity” can also depend on the application. For example, it is reported that low intensities in the range of I_SATA_ = 0.03‐0.5 W/cm^2^ can be beneficial for bone healing, whereas I_SATA_ > 0.5 W/cm^2^ (which is considered high intensity) can be detrimental.^109^, ^110^, ^111^ LIUS can be administered continuously (which we will term cLIUS), or in pulses (commonly referred to as LIPUS). As of yet, there is insufficient literature to distinguish the effects of continuous vs pulsed LIUS, so we will review them here together. LIUS has long been used for the healing of bone fractures, most likely by affecting the behavior of osteoblasts.^32^, ^112^ A few studies have investigated the mechanotransduction pathways activated by LIPUS. Yoon et al found that focused LIUS opens connexin‐43 (Cx43) hemichannels on the plasma membrane, releasing ATP into the extracellular space. Extracellular ATP binds P_2_Y_1_ purinergic receptors to activate phospholipase C, which produces the secondary messenger inositol triphosphate (IP_3_) and the release of intracellular Ca^2+^ stores.^68^ Parts of this proposed mechanism have also been weakly supported by previous studies, such as the involvement of CX43^69^ and release of extracellular ATP.^80^ The release of intracellular Ca^2+^ has interesting parallels with the mechanisms of pFUS as discussed above **(**Figure 3A**)**.

### 4.1 In vitro effects of LIUS

Most studies on LIUS have applied it to cultured MSCs in vitro (Figure 3B). Several studies have demonstrated that both cLIUS^74^ and LIPUS^73^ increase MSC proliferation through the activation of MAPK/ERK and PI3K/Akt signaling, resulting in upregulation of various cyclins. LIPUS also enhances in vitro MSC migration and upregulates adhesion molecules like CXCR4, integrin‐1β, and CCR2.^46^, ^65^ LIUS also has a well‐documented influence on MSC differentiation. There are some inconsistencies, however, regarding how it affects MSCs in normal culture conditions. Kusuyama et al found that LIPUS enhances stemness, in part by upregulating the stem cell factor Nanog.^80^ Lai et al, however, found that LIPUS pushes MSCs toward an osteogenic fate,^81^ whereas Lee et al found cLIUS to push MSCs to a chondrogenic fate.^79^ These discrepancies may arise due to different ultrasound settings, culture conditions, or cell source. What has consistently been demonstrated, however, is that when MSCs are already induced toward a certain fate, LIUS enhances differentiation toward that lineage. For MSCs cultured in osteogenic induction media, LIPUS enhances the expression of osteogenic markers such as COL1, COL10, ALP, BMP2, OCN, OPG, OPN, and OSX, as well as calcium deposition.^66^, ^72^, ^81^, ^82^, ^83^, ^84^, ^85^, ^89^, ^90^ When MSCs are cultured in chondrogenic medium, cLIUS and LIPUS enhance the expression of chondrogenic genes like COL2, aggrecan, SOX9, TIMP2, and the production of glycosaminoglycans.^78^, ^79^, ^81^, ^91^, ^92^, ^93^, ^100^ Following adipogenic induction, LIPUS enhances MSC differentiation into adipocytes, as indicated by increased expression of PPARγ, APN, and FABP4.^89^, ^94^ At least one study has shown that when MSCs are cultured in HGF, ultrasound accelerates differentiation into hepatic fates, based on the expression of AFP, ALP, and CK18,^75^ though this study used higher intensity sonication than is typical of LIPUS. Finally, a few studies have shown that cLIUS^95^ and LIPUS^76^ enhance the differentiation of MSCs into neural fates, increasing secretion of neurotrophic factors like BDNF and NGF,^76^ as well as the expression of neural markers (MAP2, ND1, NF‐L, tau) and calcium channels (CACNA1).^95^ Indeed, in a rat model of spinal cord injury, administering MSCs that had been prestimulated with LIPUS better improved locomotor function and reduced cavity formation.^76^ In addition, in a mouse model of stroke, cLIUS‐stimulated MSCs better reduced infarct area compared with unstimulated MSCs.^95^ However, it is dubious that these therapeutic effects are the result of MSCs differentiating into neurons to support regeneration; it is more likely that LIUS enhanced their production of therapeutic molecules.

A few studies have looked into the molecular mechanism by which LIUS enhances differentiation (Figure 3B). Chiu et al demonstrated that LIPUS‐mediated enhancement of osteogenesis is due to upregulation of soluble RANKL,^83^ though this result seems to conflict with other studies showing that RANK signaling in MSCs inhibits osteogenesis.^113^ Xia et al showed that LIPUS enhances chondrogenesis by activating mechanotransduction pathways through integrin‐mTOR signaling,^67^ whereas Kusuyama et al demonstrated LIPUS‐mediated enhancement of both osteogenesis and adipogenesis functions through the Rho‐associated protein kinase (ROCK), which subsequently phosphorylates the Cot/Tpl2 kinase, which is then responsible for activating the MAPK pathway.^89^ Given that Rho plays a large role in regulating the cytoskeleton, it may be an effector of LIUS‐induced mechanotransduction.

### 4.2 In vivo effects of LIUS

Although most studies have applied LIUS to cultured MSCs in vitro, a few have applied it to the actual target tissue in vivo. Two studies have used animal models of closed bone fractures, administering LIPUS to the fracture site following an infusion of MSCs.^46^, ^96^ Both found that the combined treatment enhances fracture healing and mechanical strength compared with MSCs alone. However, although one study suggested that this enhancement was due to increased MSC homing from upregulated CXCR4/SDF‐1 signaling,^46^ the other study actually found no increase in the number of MSCs at the fracture site.^96^

Hui et al used LIPUS to enhance spinal fusion in a rabbit model.^97^ They implanted a scaffold with or without embedded MSCs and administered LIPUS to the area post‐operation. MSCs + LIPUS had the highest rate of spinal fusion (86%), compared with MSCs alone (14%) or scaffold alone (0%). The combined treatment increased new bone volume and showed the greatest extent of osteochondral bridging. Another study showed that LIPUS sonication enhances the ability of MSCs to repair both bone and cartilage in a rat model of osteochondral defect.^98^

## 5 EXTRACORPOREAL SHOCK WAVE THERAPY

ESWT uses high‐amplitude acoustic waves to deliver mechanical forces to the tissue. In ESWT, a shockwave is induced by transmitting high‐pressure ultrasound wave (generally a 1 microsecond spike at roughly 50 MPa).^29^ ESWT is traditionally used in kidney stone lithotripsy^33^ and in physical therapy.^114^ Data suggest that the underlying mechanism of ESWT is based on its ability to reduce inflammatory reactions, enhance angiogenesis, suppress oxidative stress and apoptosis, and upregulate SDF‐1.^51^, ^52^ This has led some groups to investigate whether it can enhance MSC‐based therapies. As was the case with LIUS, ESWT has been applied both to cultured MSCs in vitro (Figure 3B), as well as to target tissues in vivo. Though different types of ESWT exist, it is infrequently reported in studies, and thus we will treat them here collectively.

### 5.1 In vitro effects of ESWT

In vitro, there is evidence that ESWT increases MSC proliferation by activation of the MAPK pathway.^69^ Shockwaves also seem to induce MSCs to secrete more growth factors and cytokines, such as VEGF and CXCL5.^53^, ^60^, ^70^, ^77^ Indeed, the conditioned media of ESWT‐treated MSCs better enhances neurite growth and endothelial tube formation in vitro. It also increases the expression of homing factors like SDF‐1 and improves in vitro migration.^53^, ^71^ Similar to LIUS, ESWT seems to enhance the differentiation of MSCs toward osteoprogenitor cells in vitro, as evidenced by greater expression of osteogenic markers like RUNX2, BMP2, ALP, OCN, and OSX.^77^, ^86^, ^87^, ^99^ This effect may be mediated by the activation of focal adhesion kinases.^88^

### 5.2 In vivo effects of ESWT

The potential for ESWT to enhance MSC‐based therapies has been investigated in a number of organ systems. The following studies all administered ESWT to the target organ rather than cultured MSCs. In the central nervous system, Lee et al used a rat model of spinal cord injury to demonstrate that ESWT enhances MSC engraftment by enhancing the SDF‐1 gradient.^54^ Chen et al administered ESWT to a rat model of brain death‐induced injury.^59^ They found that MSCs + ESWT was superior to either individually, decreasing circulating inflammatory cells, reducing apoptosis (decreased cleaved caspase‐3, PARP, and mitochondrial BAX), reducing inflammatory markers (TNF‐α, NF‐κB, MMP9, IL‐1β), and alleviating oxidative stress (decreased NOX1, NOX2, and p‐H2AX, and increased SIRT1, SIRT3, and mitochondrial Cytochrome C).

In a rat model of a segmental femoral defect, ESWT applied to the bone defect was able to increase MSC homing.^60^ The MSCs were found to differentiate into both osteoblastic and chondrocytic fates. Furthermore, ESWT increased the local expression of both TGFβ and VEGF, which likely played chemotactic and mitogenic roles.

ESWT has also been used in the context of myocardial infarction. Fu et al administered ESWT to infarcted heart tissue, resulting in increased vessel density and reduced fibrosis. The sonicated tissue also showed lower levels of markers for oxidative stress and apoptosis.^52^ In a porcine model of myocardial infarction, Sheu et al demonstrated that MSCs + ESWT is superior to either treatment alone at increasing heart function, reducing infarct size, and lessening left ventricular remodeling.^53^ At the site of infarction, both individual and combined treatment reduced inflammatory and oxidative stress biomarkers (MMP9, TNF‐α, NF‐κB, NOX1, NOX2), increased angiogenesis markers (CXCR4, SDF‐1, VEGF, eNOS, CD31), and decreased apoptosis markers (BAX, cleaved Casp3, PARP). Interestingly, applying ESWT to MSCs in vitro seemed to increase their expression of homing and angiogenic factors, including SDF‐1, CXCR4, VEGF, and angiopoietin.

ESWT was applied in combination with MSCs in a rat model of acute ischemia‐reperfusion muscle injury. This combination of MSC + ESWT markedly improved muscle repair more so than either treatment alone.^35^ After 7 days, both the individual and combined treatment condition exhibited decreased fibrosis (decreased TGF‐β, p‐SMAD3, and increased BMP2, p‐SMAD1/5), reduced inflammation (decreased ICAM‐1, MMP9, TNF‐α, NF‐κB, RANTES, TLR2, TLR4, IL‐1β), less DNA‐damage (decreased p‐H2AX), decreased apoptosis (decreased cytoplasmic cytochrome c, cleaved Casp3, PARP, and increased Bcl‐2), lower oxidative stress (decreased NOX1, NOX2), and increased angiogenesis in the damaged muscle. ESWT also upregulated SDF‐1 and VEGF in the muscle.^51^

Various diabetic complications have also been shown to be improved by MSCs and ESWT. In rat models of diabetic erectile dysfunction, MSCs + ESWT to the penile tissue was shown to enhance the number of MSCs engrafted in the corpus cavernosum and improve erectile function.^55^, ^56^ ESWT not only induced VEGF expression in MSCs but also increased the expression of homing molecules in the penile tissue, such as SDF‐1 and PECAM. The upregulation of PECAM is interesting, as it is a well‐known molecule in leukocyte transmigration; whether it has the same role in MSC transmigration has yet to be shown. In a rat model of diabetic bladder dysfunction, MSCs + ESWT to the bladder improved MSC engraftment and voiding function.^71^ There was increased expression of SDF‐1 and VEGF, as well as the neural growth factor NGF, which could collectively improve the vascularization and innervation of the bladder.

Even with a wealth of preclinical data, there is currently no mechanistic understanding of how ESWT enhances regeneration and how it might synergize with MSC‐based therapies. More rigorous molecular studies are necessary to understand what pathways are being activated in the tissue immediately following ESWT administration.

## 6 ULTRASOUND‐MEDIATED MICROBUBBLE DESTRUCTION

Many groups have used adjuvants alongside ultrasound to enhance its therapeutic effects. UMMD has been a popular area of research for improving MSC homing. Microbubbles are 1‐10 μm gas bubbles traditionally used as a contrast agent for ultrasound imaging, though they can also be used to enhance therapeutic effects of ultrasound, such as increasing the porosity of tissue. Sound waves applied to microbubbles can generate fluid microjets, shock waves, streaming, and cavitation forces that give rise to shear stresses on the cellular membrane that disrupt endothelial linings and increase vascular permeability.^115^, ^116^, ^117^, ^118^ The increased permeability has been exploited to improve drug and gene delivery to various tissues, including the heart^119^ and across the blood‐brain barrier.^45^, ^120^, ^121^ The same principle has been applied to increase MSC homing. It is unclear whether the type of ultrasound influences the efficacy of UMMD; most studies have used pFUS, though some have used cFUS,^48^, ^49^ LIUS,^58^ or even DUS.^36^, ^63^

Several groups have tested UMMD in combination with MSCs to treat cardiac damage resulting from myocardial infarction. Clinically, the combination of MSCs plus UMMD appears to improve heart function, decrease infarct area, and increase capillary density more than either treatment alone.^44^, ^48^, ^49^, ^50^, ^62^, ^63^, ^102^, ^103^ Following UMMD, more MSCs were found in the infarcted myocardium,^49^, ^102^ probably through a combination of increased vascular permeability and alterations to the microenvironment. Indeed, UMMD causes an upregulation of SDF‐1 at the target tissue and CXCR4 on the MSCs, which would promote their activation.^48^, ^49^, ^50^ Adhesion molecules are also found to be upregulated in the sonicated tissue (VCAM‐1, ICAM‐1),^49^, ^50^, ^62^, ^63^ growth factors (VEGF, FGF),^48^, ^50^, ^62^, ^63^ and cytokines (IL‐1β, IL‐4, IL‐6, MCP1, TNF‐α),^44^, ^50^ which would further promote MSC homing and facilitate regeneration. In a rat model of stroke, UMMD has been shown to enhance MSC homing to the brain twofold, compared with either MSCs alone or ultrasound without microbubbles.^101^ MSCs + UMMD better reduced infarct volume, cerebral edema, and the neurological severity score, though no molecular mechanisms were investigated.

In the prostate, UMMD has been shown to enhance MSC homing in a rat model of chronic bacterial prostatitis, reducing inflammatory cell infiltration and fibrous tissue hyperplasia.^58^ The combined MSC + UMMD treatment reduced TNF‐α and IL‐1β levels in the prostate, reflecting reduced inflammation; the individual treatments, however, did not result in such a reduction.

In the kidney, UMMD has been used to enhance MSC homing in a mouse model of diabetes.^36^ The sonication resulted in increased local expression of cytokines (IL‐1α, IL‐2, IL‐3, IFN‐γ, TNF‐α, MCP‐1), integrins (VCAM‐1), selectins (E‐selectin), and trophic factors (SDF‐1, VEGF). No signs of kidney damage were observed resulting from UMMD. In a rat model of AKI, UMMD was able to increase MSC homing to the kidney 2.4‐fold compared with MSCs alone. It also upregulated integrins (ICAM‐1) and growth factors (HGF, EFG) in the sonicated tissue and reduced histological signs of kidney damage.^61^ Wu et al developed microbubbles loaded with SDF‐1.^105^ Infusion of the SDF‐1‐loaded microbubbles followed by focused ultrasound to the kidney enhanced MSC homing 1.8‐fold compared with normal microbubbles and 6.6‐fold over ultrasound alone.

UMMD appears to elicit a greater therapeutic response than ultrasound alone. However, it does come with an inherent problem: microbubble cavitation disrupts tissue integrity and cell membranes and can thus cause hemorrhage.^122^, ^123^, ^124^ Though some studies report finding no evidence of such micro‐hemorrhages,^125^, ^126^ UMMD in its current state is still faced with some safety concerns. There are few studies specifically seeking to improve the safety of UMMD, which may depend on various parameters of the sound waves used for cavitation.

## 7 DISCUSSION

Here we have presented a comprehensive review of the methods by which ultrasound has been leveraged to enhanced MSC‐based therapies. A variety of strategies exist: whether to sonicate the cultured MSCs or the target tissue, whether to utilize adjuvants like microbubbles, and whether to use low‐ or high‐intensity sound waves. The large number of studies in this field has implicated several potential pathways by which sound waves exert their biological effects through mechanotransduction; however, further studies still need to be performed to completely understand the exact mechanisms involved. Indeed, future studies need to systematically investigate the immediate and long‐term responses in sonicated tissue as a function of ultrasound intensity.

### 7.1 The need for standardization in ultrasound parameter reporting

One of the most pressing needs in the field is standardization in ultrasound parameter reporting. The simple categorization scheme shown in Figure 1 is insufficient; for instance, the intensities used under the umbrella of “pFUS” span orders of magnitude. More problematic is that many studies do not report intensity parameters, and of the ones that do, most do not state what kind of intensity was measured. This trend is troubling for the reproducibility of studies in this field and makes meta‐analyses impossible. We therefore recommend that all studies using therapeutic ultrasound to optimize cell therapy report temporal average intensities (I_SATA_ and I_SPTA_), because the induced bioeffects in these studies are dependent on the temporal application of ultrasound. I_SATA_ describes the average acoustic power applied to tissue over time. I_SPTA_, although perhaps less useful, describes the maximum power applied to the tissue over the course of the treatment. MI (or frequency and peak negative pressure) should also be reported, as it indicates the extent of mechanical bioeffects. Furthermore, all studies utilizing microbubbles should additionally report the temporal peak intensities (I_SATP_ and I_SPTP_). Because bubbles are responsive to the instantaneous pressure, the temporal peak intensities will be informative of the therapeutic effect of microbubbles.

With improved reporting, researchers in the field will be better equipped not only to reproduce studies but also to expand upon, and further refine, therapeutic efficacy. Ultrasound as a method to improve MSC‐based therapies is a vast area for further research that is frequently emerging with new data to improve its utilization. Optimizing this clinical strategy would be a boon to the field of regenerative medicine, broadly boosting the effectiveness of therapeutics in applications from immune modulation to regeneration.

## CONFLICT OF INTEREST

The authors declared no potential conflicts of interest.

## AUTHOR CONTRIBUTIONS

D.D.L.: conception and design, administrative support, collection and/or assembly of data, data analysis and interpretation, manuscript writing; M.U.: conception and design, collection and/or assembly of data; W.C.: conception and design, financial support; J.J.D.: conception and design, data analysis and interpretation, manuscript writing, final approval of manuscript; A.S.T.: conception and design, financial support, administrative support, final approval of manuscript.

## Data Availability

Data sharing is not applicable to this article as no new data were created or analyzed in this study.
